# Features of Flemish Emerging Adults and their Association with Demographic Markers

**DOI:** 10.5334/pb.514

**Published:** 2020-02-20

**Authors:** Laura Mattys, Koen Luyckx, Ilse Noens, Dieter Baeyens

**Affiliations:** 1 Faculty of Psychology and Educational Sciences, KU Leuven, Leuven, BE

**Keywords:** emerging adulthood, young adult, Flanders, development, factor analysis, adolescent, identity

## Abstract

As the Inventory of Dimensions of Emerging Adulthood (IDEA) was developed to assess features of emerging adulthood, international differences have been observed between emerging adults over the world. To assess the IDEA’s applicability to emerging adults in Flanders, a Dutch translation was administered to 489 participants between 17 and 26 years old, and exploratory and confirmatory factor analyses were performed. Important latent factors in the version used in the United States of America, such as focus on others, were not observed. Four subscales were created to reflect psychological features of our sample, combining elements of both Arnett’s theory of emerging adulthood and neo-Eriksonian theories of identity development. A new subscale, reflecting commitment and responsibility, was more present in older participants and employed participants, and associated with well-being. Finally, results suggested that employment, as well as place of residency, and romantic relationships significantly influence psychological development during young adulthood.

## Introduction

### Assessing features of emerging adulthood

Arnett (2000) has described psychological features of individuals between 18 and 25 years old in a developmental phase that he named *emerging adulthood*. Previously, he reported that young Americans conceptualize maturity as accepting responsibility for one’s self and making independent decisions, along with becoming financially independent (Arnett, 1998). Consequently, Arnett (2000) describes emerging adulthood in terms of five core features: individuals are focused on themselves (*self-focus*), as they are not tied to the routine of school and not constrained by marriage, children, or a career yet. They also explore who they are (*identity exploration*), are optimistic about, and experiment with possibilities they have for their futures (*experimentation/possibilities*). They situate themselves in a phase between adolescence and adulthood (*feeling in-between*), and also experience stress and instability because of changes in residency, relationship status, and occupation (*negativity/instability*; Arnett, 2000). To measure Arnett’s five features of emerging adulthood, the Inventory of Dimensions of Emerging Adulthood (IDEA; Reifman, Arnett, & Colwell, 2007) was developed, using a sample of predominantly white female college students and their non-college acquaintances. The features are represented in five subscales, with the addition of one subscale, *other-focus*, measuring the opposite of *self-focus*. Considering indicators of well-being, Reifman et al. (2007) found that more *experimentation and possibilities* and higher *other-focus* were linked to higher life satisfaction. *Negativity and instability* coincided with lower life satisfaction (Reifman et al., 2007).

Since its publication, the IDEA questionnaire and its application have been studied internationally using exploratory (and sometimes also confirmatory) factor analyses (Atak & Çok, 2008; Baggio, Iglesias, Studer, & Gmel, 2015; Dutra-Thomé, 2013; Hill, Lalji, van Rossum, van der Geest, & Blokland, 2015; Leontopoulou, Mavridis, & Giotsa, 2016; Lisha et al., 2014; Pérez, Cumsille, Loreto, & Martinez, 2008). Appendix 1 provides an overview of how items were redistributed across subscales in each study. Overall, the subscale *negativity and instability* is supported across different studies. The concepts of *feeling in-between* and *experimentation and possibilities* seem especially relevant in western populations. Most items in *identity exploration* also seem to measure the intended concept across studies, although some items were found to load on other latent constructs as well. Finally, items originally meant to assess *self-focus* and *other-focus* were often reallocated to other constructs, and in one case even part of the same latent factor. Other authors have also used the IDEA, but adapted their version to the point where results were not comparable to the original anymore (Arias & Moreno Hernandez, 2007; Macek, Bejček, & Vaníčková, 2007), only used the subscale *negativity and instability* (Musante, 2010; Luyckx, de Witte, & Goossens, 2011), or simply translated the tool without reporting further analyses (Adamek, Dreher, & Mayr, 2004, in Sirsch, Dreher, Mayr, & Willinger, 2009; Facio, Resett, Micocci, & Mistrorigo, 2007).

Besides potential issues with some of the translations used, the variability of these results may demonstrate the cultural dependency of these core features of emerging adulthood as defined by Arnett (2000). Therefore, we evaluated the applicability of the IDEA in a Flemish sample. As content analysis (Ravert, Stoddard, & Donnellan, 2018) noted that the most frequently mentioned limitation in studies focusing on emerging adults is the use of a college sample, we included different demographic profiles (secondary education students, higher education students, employed participants, and unemployed participants) in the age range of 17 to 26 years old in the present study.

The main goal of this study is to describe psychological features of emerging adults in Flanders starting from Arnett’s theory of emerging adulthood, as operationalized in the IDEA. In a first phase, we evaluated the IDEA in a Flemish sample, using exploratory and confirmatory factor analysis. We hypothesized that the original factor structure of the IDEA would not be fully replicated in a Belgian sample, and, based on previous research, specifically expected the subscales *identity exploration, self-focus*, and *other-focus* not to be fully replicated. In a second phase, we examined the external validity of the obtained subscales by relating them to demographic markers such as age, gender, occupation, place of residence, and relationship status, as well as indicators of well-being.

## Method

### Participants

The sample was recruited by master thesis students in Flanders (the Dutch speaking part of Belgium) using snowball sampling starting from their own social network. Two inclusion criteria considering occupation and age were employed. For occupation, we purposefully included a variety of participants, including secondary education students, higher education students, employed participants, and unemployed participants who were not enrolled in any educational program. For age, we recruited participants between 17 and 26 years old, to be able to study trajectories of growth in the context of a larger longitudinal project. An ideal sample size of 500 participants was determined to reach statistical power of 0.990 for these growth trajectory analyses. A total of 489 participants between 17 and 26 years old (Table 1) filled out an online survey in Dutch.

**Table 1:** Participant characteristics.

Occupation	Relationship status	*n* (male/female)	mean age (years)	SD age (years)

Secondary student		66 (27/39)	17.35	0.74
	Single	38 (17/21)	17.37	0.88
	In a relationship	25 (8/17)	17.36	0.49
	Complicated	3 (2/1)	17.00	0.00
Higher education student		268 (114/154)	20.92	1.93
	Single	135 (58/77)	20.53	1.99
	In a relationship	128 (53/75)	21.21	1.72
	Complicated	5 (3/2)	24.00	1.41
Employed		141 (64/77)	23.77	1.80
	Single	41 (24/17)	22.98	2.19
	In a relationship	98 (40/58)	24.08	1.52
	Complicated	2 (0/2)	24.50	0.71
Unemployed		14 (7/7)	23.36	1.50
	Single	5 (2/3)	22.80	1.94
	In a relationship	9 (5/4)	23.67	1.23
	Complicated	0 (0/0)	/	/
		489 (212/277)	21.33	2.676

### Measures

#### Demographic markers

Demographic variables included gender, date of birth, occupation (in secondary education, in higher education, employed, or unemployed), relationship status (in a relationship, single, or ‘it’s complicated’ when they were not in a committed relationship, but did not consider themselves single, either), and place of residence (living with parents, in student dorm, alone, with partner, or non-student living with roommates).

#### Features of emerging adulthood

A Dutch translation of the Inventory of Dimensions of Emerging Adulthood (IDEA; Reifman, Arnett, & Colwell, 2007) previously used by Hill et al. (2015) was administered to assess dimensions of emerging adulthood. It consisted of 30 items. For each item, participants are asked to indicate their level of agreement with a statement on a four-point Likert scale (1 = *strongly disagree*, 4 = *strongly agree*). Scores are represented in six subscales, which correspond with the five described dimensions of emerging adulthood (*identity exploration, experimentation/possibilities, negativity/instability, self-focus*, and *feeling in-between*), and one counterpart (*other-focus*). Higher scores indicate more endorsement of each dimension. When publishing the original IDEA and testing its psychometric properties on a Texan sample, reported internal consistency was reasonable, with α between 0.70 and 0.85 for each subscale. Test-retest reliability of the subscales ranged from 0.64 to 0.76 over a one-month interval, except for *feeling in-between*, which only showed a reliability of 0.37 (Reifman et al., 2007).

#### Indicators of well-being

To assess aspects of well-being, the Quality of Life-BREF questionnaire (WHOQoL-BREF; WHO, 1996) and Revised SCL-90 Symptom checklist (SCL-90-R; Arrindell & Ettema, 2003) were administered. The World Health Organization defines quality of life (QoL) as “*an individual’s perceptions of their position in life in the context of the culture and value systems in which they live and in relation to their goals, expectations, standards and concerns*” (The WHOQOL Group, 1995: 1405). As such, it is a subjective construct, rooted in an individual’s experiences and thoughts, instead of a normative checklist, assessing standard indicators of health. The WHOQoL-100 questionnaire was simultaneously developed in fifteen international field centers by the World Health Organization, as an assessment of quality of life that can be applied cross-culturally. The shortened version used in this study contains 26 questions, which participants answer in a Likert format, ranging from 1 (very poor/not at all/very unsatisfied/very bad) to 5 (very good/completely/very satisfied/very well). The first question explicitly asks “*How would you rate your quality of life?*” and is examined separately in this study. A cross-cultural study in 23 countries demonstrated the WHOQoL-BREF’s good to excellent internal consistency, discriminant validity, and construct validity (Skevington, Lotfy, O’Connell, & The WHOQOL Group, 2004).

A Dutch translation (de Vries & van Heck, 1996) was administered. Scores are reflected in four raw subscales, representing four domains of QoL: *physical health* encompasses topics such as pain and discomfort, activities of daily living, and sleep; the *psychological* domain includes negative and positive feelings, self-esteem, bodily image and concentration; *social relationships* contains personal relationships, social support and sexual activity; and *environment* assesses topics like financial resources, freedom, physical safety, home environment, accessibility and quality of health care, participation, pollution and transport.

The SCL-90-R was developed to identify psychological and physical symptoms of psychological problems (Derogatis, 1979). We administered a Dutch translation (Arrindell & Ettema, 2003), which has high internal consistency (α = 0.97), good construct validity, good convergent validity over similar instruments, and high reliability over two months (*r* = 0.82, n = 91) and used the total raw sum score.

### Data-analytic strategy

#### Revision IDEA

Formal testing of data distribution showed that item responses were highly skewed towards the higher side (*W* (489) = 0.603– 0.880; *p* < 0.001). In addition, a 4-point Likert scale generates ordinal data. As such, estimators using weighted least squares with means and variances adjusted based on polychoric correlations were opted for in analyses and carried out in MPlus (Muthén & Muthén, 2007). In a first step, the proposed model with six dimensions underlying the IDEA questionnaire (Reifman et al., 2007) was tested using confirmatory factor analysis. Subsequently, the latent structure of the IDEA questionnaire was examined using exploratory factor analysis, and a modified set of subscales, named IDEA-BE, was developed.

Next, the new factor structure was tested using confirmatory factor analysis. To evaluate model fit, the Chi square test statistic with corresponding degrees of freedom and level of significance was used to inform decision making, but not as criterion, as the sample exceeds 400 cases, inflating calculated values (Schumacker & Lomax, 2004). Instead we used a two-criteria strategy, including an incremental fit index (comparative fit index, CFI) and a parsimony-adjusted absolute fit index (root mean square error of approximation, RMSEA). For the CFI index, we used a cutoff of 0.90 to indicate adequate fit, and 0.95 to indicate excellent fit (Kline, 2006). For the RMSEA, we used a cutoff of 0.08 to indicate adequate fit, and 0.06 to indicate excellent fit (Kline, 2006).

#### Exploration IDEA-BE

In a second step, we explored how the new subscales in the IDEA-BE (Belgian version of the IDEA) relate to demographic markers and indicators of well-being. No participants were excluded from these analyses. Pearson correlations were used to examine correlations between the subscales, and their relationships to age, WHOQoL-BREF scores, and SCL-90-R scores. As the distribution of IDEA-BE scores was highly skewed across data (*W* = 0.914–0.979, *p* < 0.001), Kruskall-Wallis tests, a nonparametric method of analysis (Siegel, 1957), were used to compare the distributions of the subscale scores over different occupations, places of residence, and relationship statuses. We calculated *E^2^* coefficients as a proxy for effect sizes. For gender, the Mann-Whitney U coefficient was used, and ρ-values were calculated to estimate effect size. ρ-values indicate the overlap between distributions and can range from 0 to 1, where both extremes represent total separation, and 0.50 represents total overlap of the distributions.

## Results

### Revision IDEA

The latent model proposed by Reifman et al. (2007) was tested and showed a poor fit with our data (χ^2^ (390) = 1908.90, *p* < 0.001; CFI = 0.846; RMSEA = 0.083). Consequently, exploratory factor analysis was conducted. Factors were rotated using an oblique geomin rotation, as they were expected to correlate. Table 2 provides an overview of initial eigenvalues and comparative fit indices. Considering not only these, but also theoretical interpretability of rotated factor loadings, and approximation of simple structure (where an adequate number of factors load highly on each factor, and high cross-loadings are avoided where possible), the four-factor model provided an optimal solution.

**Table 2:** Initial Rotated Factor Loadings.

Item	Is this period in your life a time of…	Factors	1	2	3	4	IDEA^1^

1	many possibilities		0.662				ExpPos
2	exploration		0.758				ExpPos
3	experimentation		0.732				ExpPos
4	open choices		0.714				ExpPos
5	trying out new things		0.764				ExpPos
6	feeling adult in some ways but not others			0.496			FeelIB
7	gradually becoming an adult			0.604			FeelIB
8	unsure of having reached full adulthood			0.546			FeelIB
9	finding out who you are			0.622			IDExpl
10	separating from parents			*0.303*	*0.356*		IDExpl
11	defining yourself			0.509	0.408		IDExpl
12	planning for the future					0.627	IDExpl
13	seeking a sense of meaning			0.508			IDExpl
14	deciding your own beliefs and values			0.618	*0.360*		IDExpl
15	learning to think for yourself				0.612	*0.305*	IDExpl
16	confusion			0.411	0.542		NegInst
17	feeling restricted		*–0.307*		0.576		NegInst
18	feeling stressed out				0.919		NegInst
19	instability				0.717		NegInst
20	high pressure				0.936		NegInst
21	unpredictability				0.591		NegInst
22	many worries				0.810		NegInst
23	settling down					0.656	OtherFoc
24	responsibility for others					0.477	OtherFoc
25	commitments to others					0.499	OtherFoc
26	personal freedom		0.433				SelfFoc
27	responsibility for yourself					0.450	SelfFoc
28	optimism		0.401			–0.410	SelfFoc
29	independence					0.394	SelfFoc
30	focusing on yourself						SelfFoc

Only factor loadings larger than 0.300 are displayed. Factor loadings smaller than 0.390 are printed in cursive. ^1^ IDEA subscale = IDEA, Experimentation/possibilities = ExpPos, feeling in-between = FeelIB, identity exploration = IDExpl, negativity/instability = NegInst, other-focus = OtherFoc, and self-focus = SelfFoc.

As recommended by Worthington and Whittaker (2006), in a first step, items 10 and 30, with factor loadings lower than 0.390 were deleted, and the analysis was run again. Next, items with cross-loadings over 0.330 (items 11, 13–16, and 28) were omitted and the analysis was again repeated. The output showed that items 17 and 26 had cross-loadings above the cut-off, so they were deleted as well. Table 3 shows the final model, with factor loadings between 0.436 and 0.932 for each item.

**Table 3:** Rotated Factor Loadings Final Model.

Item	Is this period in your life a time of…	Factors	1	2	3	4	IDEA^1^

1	many possibilities		0.703				ExpPos
2	exploration		0.800				ExpPos
3	experimentation		0.800				ExpPos
4	open choices		0.729				ExpPos
5	trying out new things		0.763				ExpPos
6	feeling adult in some ways but not others			0.693			FeelIB
7	gradually becoming an adult			0.641			FeelIB
8	unsure of having reached full adulthood			0.722			FeelIB
9	finding out who you are			0.493			IDExpl
12	planning for the future					0.612	IDExpl
18	feeling stressed out				0.869		NegInst
19	instability				0.703		NegInst
20	high pressure				0.932		NegInst
21	unpredictability				0.581		NegInst
22	many worries				0.807		NegInst
23	settling down					0.724	OtherFoc
24	responsibility for others					0.460	OtherFoc
25	commitments to others					0.558	OtherFoc
27	responsibility for yourself					0.486	SelfFoc
29	independence					0.436	SelfFoc

*Only* factor loadings larger than 0.300 are displayed. ^1^ IDEA subscale = IDEA, Experimentation/possibilities = ExpPos, feeling in-between = FeelIB, identity exploration = IDExpl, negativity/instability = NegInst, other-focus = OtherFoc, and self-focus = SelfFoc.

Analogous to the concepts in the original instrument, items 1 to 5 formed the subscale *experimentation and possibilities*; items 6, 7, 8, and 9 the subscale *feeling in-between*; and items 18 to 22 were the remaining items of the subscale *negativity and instability*. A new subscale, containing items 12, 23, 24, 25, 27 and 29, was named *commitment and responsibility*.

This model was tested using confirmatory factor analysis and showed a good fit (χ^2^ (164) = 614.49, *p* < 0.001; CFI = 0.936; RMSEA = 0.069).

### Exploration modified IDEA (IDEA-BE)

#### Correlations between subscales

Pearson correlation coefficients were calculated to explore relationships between the new subscales (Table 4). Significant positive correlations were found between *experimentation and possibilities* and *feeling in-between* (*r* = 0.220, *p* < .0001), and *experimentation and possibilities* and *commitment and responsibility* (*r* = 0.160 *p* < 0.001). *Feeling in-between* correlated slightly negatively with *commitment and responsibility* (*r* = –0.107, *p* = 0.018). *Negativity and instability* correlated significantly only with *feeling in-between* (*r* = 0.318, *p* < 0.001).

**Table 4:** Pearson Correlation Coefficients.

Subscale correlations						

Subscale^1^	ExpPos	FeelIB	NegInst	Commit		

ExpPos						
Pearson’s *r*	/	0.220	–0.40	0.160		
*p*-value	/	<0.001	0.372	<0.001		
FeelIB						
Pearson’s *r*		/	0.318	–0.107		
*p*-value		/	<0.001	0.018		
NegInst						
Pearson’s *r*			/	–0.056		
*p*-value			/	0.213		
**Age**						

**Subscale**^1^	**age (years)**					

ExpPos						
Pearson’s *r*	–0.159					
*p*-value	<0.001					
FeelIB						
Pearson’s *r*	–0.216					
*p*-value	<0.001					
NegInst						
Pearson’s *r*	–0.193					
*p*-value	<0.001					
Commit						
Pearson’s *r*	0.280					
*p*-value	<0.001					
**Well-being**						

**Subscale**^1^	**subjective**	**physical**	**psychological**	**social**	**environment**	**SCL**

Pearson’s *r*	0.066	0.168	0.131	0.131	0.127	–0.110
*p*-value	0.198	0.001	0.011	0.011	0.013	0.033
FeelIB						
Pearson’s *r*	–0.163	–0.154	–0.225	–0.125	–0.027	0.159
*p*-value	0.001	0.003	<0.001	0.014	0.605	0.002
NegInst						
Pearson’s *r*	–0.314	–0.374	–0.367	–0.258	–0.258	0.460
*p*-value	<0.001	<0.001	<0.001	<0.001	<0.001	<0.001
Commit						
Pearson’s *r*	0.101	0.161	0.252	0.233	0.141	–0.139
*p*-value	0.048	0.002	<0.001	<0.001	0.006	0.007

^1^ Experimentation and possibilities = ExpPos, feeling in-between = FeelIB, negativity and instability = NegInst, commitment = Commit, WHOQOL subjectively reported quality of life = subjective, WHOQOL physical quality of life = physical, WHOQOL psychological quality of life = psychological, WHOQOL social quality of life = social, WHOQOL environment quality of life = environment, SCL-90 total raw sum score = SCL.

#### Age

Pearson correlation coefficients were calculated between each subscale and age in years (Table 4). *Experimentation and possibilities, feeling in-between*, and *negativity and instability* were negatively correlated with age. The new subscale *commitment and responsibility*, however, was positively correlated with age.

#### Gender

**Figure 1:**
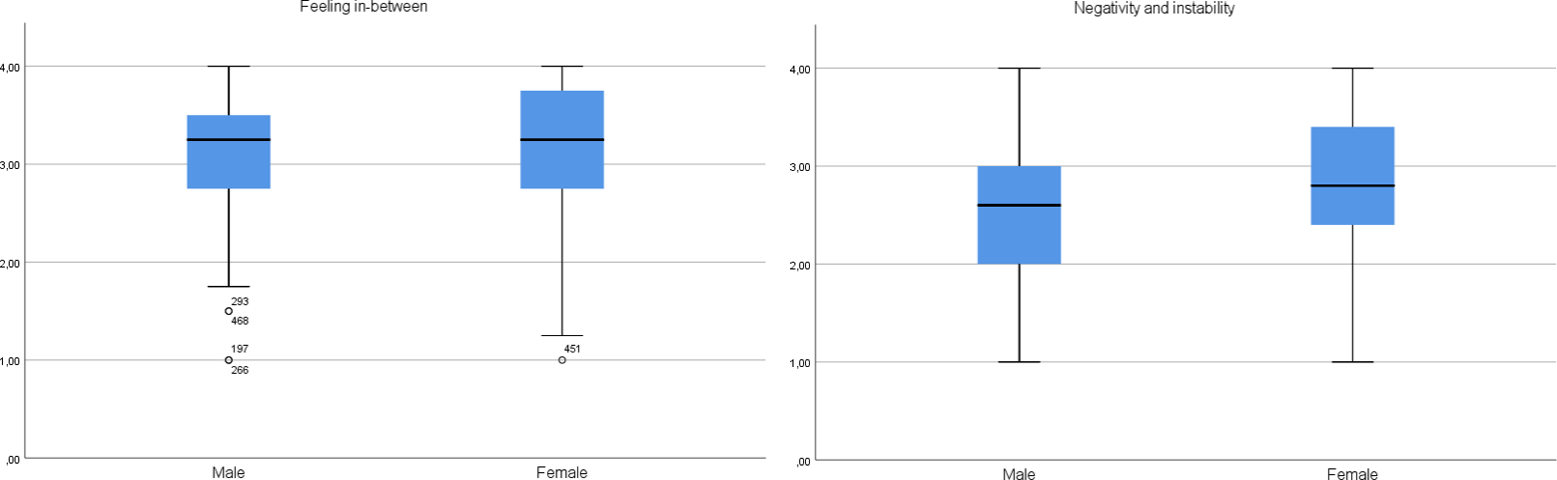
Boxplots *of feeling in-between* and *negativity and instability* scores by gender.

Scale scores for *experimentation and possibilities* and *commitment and responsibility* were not significantly differently distributed between male and female participants (U = 32 194.500, *p* = 0.064, U = 26 580.000, *p* = 0.071, respectively). For *feeling in-between* and *negativity and instability*, females more often reported higher scores than male participants (U = 25 174.500, *p* = 0.006, ρ = 0.429; U = 23 236.500, *p* < 0.000, ρ = 0.396, respectively, Figure 1). When taking into account the ρ – values, however, we notice that the distributions of *feeling in-between* still largely overlap and the significant result thus is not informative of an interpretable difference between both genders.

#### Occupation

**Figure 2:**
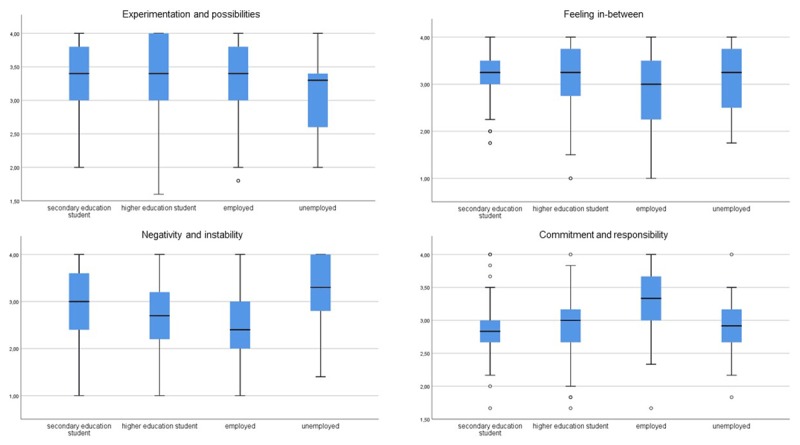
Boxplots of IDEA-BE subscale scores by occupation.

Kruskal-Wallis tests showed significant differences in distributions of all subscales between secondary education students, higher education students, employed participants and unemployed participants for all subscales (*H*(3) = 8.783–67.300, *p* < 0.001, *E^2^* = 0.018–0.138; Figure 2).

#### Place of residence

**Figure 3:**
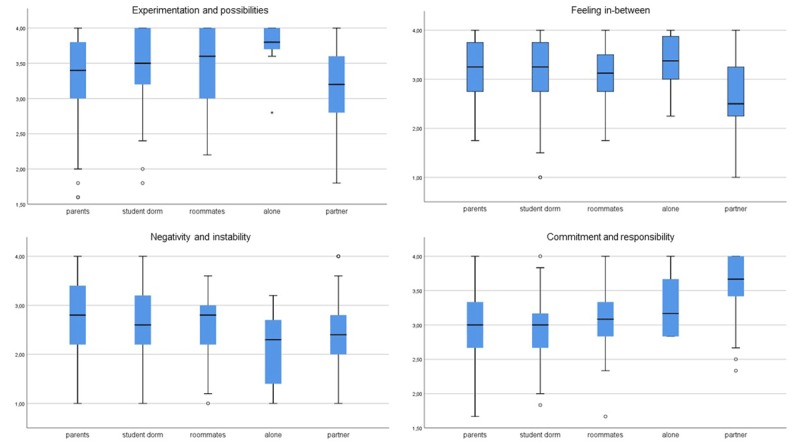
Boxplots of IDEA-BE subscales by place of residence.

Kruskal-Wallis tests demonstrated different distributions of all subscales between participants living with their parents, in student dorms, alone, with their partners, or in shared housing (*H*(4) = 10.965–61.693, *p* < 0.05, *E^2^* = 0.022–0.126; Figure 3).

#### Relationship status

For relationship status, no significantly different distributions were detected between participants in *experimentation and possibilities* (*H*(2) = 1.231, *p* = 0.540), *feeling in-between* (*H*(2) = 1.849, *p* = 0.397), and *negativity and instability* (*H*(2) = 4.450, *p* = 0.108). However, for *commitment and responsibility* (*H*(2) = 57.409, *p* < 0.001, *E^2^* = 0.118), participants in a relationship (mean rank 290) ranked much higher than those who were single (mean rank 193). Participants who indicated “it’s complicated” ranked in between the two other groups (mean rank 204).^1^

#### Well-being

Pearson correlations were calculated between IDEA-BE subscale scores and indicators of well-being (Table 4). Directly reported quality of life through the first question of the WHOQoL-BREF questionnaire was negatively correlated with *negativity and instability*, and *feeling in-between*. A small positive correlation was found with *commitment and responsibility*, and no significant correlation was found with *experimentation and possibilities*. As for the WHOQoL-BREF subscales of physical health, psychological health, social relationships, and environment, *experimentation and possibilities* was slightly positively correlated to all of them. *Feeling in-between* was slightly negatively correlated to all subscales except environment. Reported *negativity and instability* was negatively correlated to physical and psychological health, social relationships, and environment. Finally, *commitment and responsibility* showed a positive correlation with all subscales, the strongest of which with psychological health, followed by social relationships, physical health, and environment.

Symptomatology as measured with the SCL-90 questionnaire was correlated with *negativity and instability*, slightly correlated with *feeling in-between*, and slightly negatively correlated with *commitment and responsibility* and *experimentation and possibilities*.

## Discussion

### Theoretical background of IDEA-BE subscales

As hypothesized, the original latent structure of the IDEA questionnaire did not fit the response patterns of our Flemish sample. The subscales were restructured, resulting in a modification (IDEA-BE) with four subscales; *experimentation and possibilities, feeling in-between, negativity and instability*, and *commitment and responsibility*. The first three clearly resonate with Arnett’s original descriptions of core developmental features of emerging adulthood, the fourth seems to add a highly relevant task for youth on their way to adulthood.

Participants with higher scores on the first subscale, *experimentation and possibilities*, described this period in their lives as a time of “many possibilities”, “exploration”, “open choices”, and “trying out new things”. This subscale only consists of items of the original IDEA subscale, and thus could be best explained by Arnett’s definition of the core feature (2000). It indeed has proven to be a relatively robust subscale across samples from different countries (Dutra-Thomé, 2013; Hill et al., 2015; Lisha et al., 2014; Pérez et al., 2008).

In the subscale *feeling in-between*, Arnett’s feature of feeling in-between adolescence and adulthood is clearly resonated in the items “gradually becoming an adult”, “not sure whether you have reached full adulthood”, but is also extended with the item “finding out who you are”, an item reflecting his concept of *identity exploration* in the original IDEA. In Turkey, Greece, Chili, and for at-risk Californian youth and Antillean young adults in The Netherlands as well, items from the subscale *feeling in-between* also coincided in one factor with items originally intended to assess *identity exploration*. The items convey a sense of being in-between two stages, insecurity about one’s identity, and the perception of not having reached the adult status yet. This phase in between adolescence and adulthood has been extended by changing social-economical standards and normative expectations, leaving more time to question oneself and postpone commitments, but also eliciting insecurity (Côté, 2006). In fact, this subscale was the only one to correlate positively with *negativity and instability*.

Participants with higher scores on the subscale *negativity and instability* report “feeling stressed out”, “instability”, “high pressure”, “unpredictability”, and “many worries”. Throughout various international studies, this original subscale has been replicated the most consistently (Atak & Çok, 2008; Baggio et al., 2015; Dutra-Thomé, 2013; Hill et al., 2015; Leontopoulou et al., 2016; Lisha et al., 2014; Pérez et al., 2008).

The last subscale consists of the items “planning for the future”, “settling down”, “responsibility for others”, “commitment and responsibility to others”, “responsibility for yourself” and “independence”, and was labeled *commitment and responsibility*, as these items reflect a sentiment of taking up responsibilities to both self and others, and committing to these. It consists of items originally intended to measure two separate constructs, *self-focus* and *other-focus*. This suggests that for Flemish young adults, experienced independence does not diminish the capacity to commit to other people, but that settling down, committing to others, and carrying responsibility for others are an essential part of gaining independence and working towards the future. In a previous study involving Flemish youth, Luyckx, Klimstra, Duriez, Van Petegem, and Beyers (2013) found that commitment processes indeed are more present in older participants. International research, as well, suggests that focus on oneself does not have to contradict orientation towards others, and it is also not a separate subscale: in all but one IDEA replication study (Hill et al., 2015), items intended to assess *self-focus* belonged to latent factors that also represented aspects of *experimentation and possibilities, identity exploration*, and *feeling in-between*. In Reifman’s (2007) introduction of the IDEA, it was not specified why the subscale *other-focus* was added as a counterpart to *self-focus*, and not reported how both related to each other and the other subscales. Accordingly, its status as a separate feature of emerging adulthood is scarcely supported.

In his earlier work, Arnett (1998) described how young Americans viewed accepting responsibility for one’s self and the capacity to make independent decisions, along with financial independence, as important criteria of maturity. He also noted that marriage ranked much lower in the criteria for adulthood, both compared to other cultures and to Americans in the past. Settersten, Ottusch, and Schneider (2015), however, pointed out that young adults postpone marriage precisely because it is very important to them, and that the building of an adult identity is a process, in which no single experience renders one an adult. Instead, it is a larger cluster of events and the gradual accumulation of experiences that eventually render one an adult in the eyes of oneself and others. Traditional roles, particularly marriage, now initiate a new phase in this process, rather than start it. This is clearly reflected in our results, where future-oriented, lasting decisions are associated with both independence and commitments to others, and this subscale relates negatively to *feeling in-between*. Similarly, Marcia (1966) theorized that during the transition to adulthood, Erikson’s (1968) developmental task of forming an ego identity is resolved by exploration of options on the one hand, and commitment to choices that define one’s identity on the other. As such, the items making up the subscale *experimentation and possibilities*, can be related to this concept of exploration, while the subscale *commitment and responsibility* seem to fit with Marcia’s concept of commitment.

### Describing young adults in Flanders

#### Exploring demographic markers

The IDEA-BE subscales are slightly negatively correlated with age, except for *commitment and responsibility*. In line with our findings, Reifman et al. (2007) reported that *experimentation and possibilities, identity exploration*, and *negativity and instability* were lower in the age group of 24 to 29 years old when compared to the group of 18 to 23 years old. In addition, they found *self-focus* to be higher in the younger group, but *other-focus* to be higher in the older group. Our subscale *commitment and responsibility*, taking items of both these original subscales, showed a slightly positive correlation with age. As such, it assesses an opposite feature of the other three subscales, which becomes more prevalent with age, as more commitments are made, a process that has previously been suggested by Luyckx, Klimstra, Schwartz, and Duriez (2013).

For gender, no interpretable effects were found, except for the subscale *negativity and instability*, where we found a small gender effect: females more frequently reported higher scores than males. This effect was not reported by Reifman et al. (2007), who used a predominantly female sample. Our results, however, correspond with prevalence rates, as the onset of anxiety and depressive disorders peaks during adolescence and young adulthood, and young adult women are three times more likely than men to experience depression and most anxiety disorders (Kessler et al., 1994; Weissman et al., 1994, 1996; Gater et al., 1998, in Altemus, Sarvaiya, & Epperson, 2014).

Results indicate that occupation is related to psychological features assessed with the IDEA-BE, albeit with small effect sizes. Of all occupational groups, higher education students most often reported higher *experimentation and possibilities* and *feeling in-between*. Secondary education students frequently reported higher *negativity and instability* and lower *commitment and responsibility*, reflecting their uncertainty and distress, as they possibly enter a period of many changes. These results resonate with the findings of Verschueren, Rassart, Claes, Moons, and Luyckx (2017), who concluded that Flemish higher education students reported more active exploration, whereas secondary education students were characterized by a low level of commitments. In comparing 6^th^ to 12^th^ grade students to higher education students and graduates, Reifman et al. (2007) reported no significant differences in *negativity and instability*, suggesting that high school students could indeed experience as much stress as their older counterparts. Employed participants are more likely to report less *feeling in-between* and high *commitment and responsibility*, reflecting their distance to the others, and their occupational commitment. In accordance, Reifman et al. (2007) found that the more hours participants worked, the greater *other-focus* they reported, a subscale of which items are included in *commitment and responsibility*. Luyckx et al. (2008) suggested that being employed is associated with higher perceived adult status, which logically coincides with less *feeling in-between*. Reifman et al. (2007) and Lisha et al. (2014) also reported higher *experimentation and possibilities* experienced by higher education students compared to age-matched non-students, and that higher levels of *experimentation and possibilities* associated with lower odds of having a job, and less hours worked by participants. In their comparisons, however, Reifman et al. (2007) and Lisha et al. (2014) did not distinguish between employed and unemployed non-students, only between hours worked or student status. In our study, unemployed participants more frequently reported especially low *experimentation and possibilities*, accompanied by high *negativity and instability*, possibly highlighting the importance of (perceived) means to experiment.

When considering place of residence, slightly different distributions were again found for all subscales. In accordance with Reifman et al.’s (2007) findings, we found that individuals living with friends and in student dorms most frequently reported highest *experimentation and possibilities*, whereas those living with their partners reported lowest. Interestingly, we also found that individuals living alone reported very high levels, but their sample size was too small to draw conclusions. Higher levels of *commitment and responsibility* were reported by individuals living alone or with their partners, whereas lower levels were reported more by participants who lived in student dorms or with their parents. In accordance, Reifman et al. (2007) found that those living with a partner/spouse scored highest on *other-focus* (of which items are included in our subscale *commitment and responsibility*) and those in a dormitory lowest.

For relationship status, no significantly different distributions were found between participants except for the subscale *commitment and responsibility*, where we observed that participants in a relationship were slightly more likely to report higher commitment than those who were single. This supports the conceptual framework behind the subscale. Considering marital status, Reifman et al. (2007) report results of two studies, where never-married individuals between 18–29 years old were compared to their age-matched engaged/married counterparts in two samples. In both samples, engaged/married participants reported lower levels of *experimentation and possibilities*, and higher *other-focus*, a subscale of which items are included in *commitment and responsibility*. Additional differences he found in *experimentation and possibilities* might be the result of having used a more extreme marker of relationship status.

#### Well-being

The IDEA-BE subscales were related to indicators of well-being. Results seem to support the concepts behind the subscales: *experimentation and possibilities* is positively associated with well-being, *feeling in-between* is linked to slightly lower well-being, *negativity and instability* is negatively related to well-being, and higher well-being is likely as *commitments and responsibilities* are taken up. In line with our findings, *experimentation and possibilities* has been found to correlate positively to life satisfaction before (Hill et al., 2015; Reifman et al., 2007). *Negativity and instability* has been related to lower life satisfaction (Hill et al., 2015; Reifman et al., 2007), and indications of depression (Hill et al., 2015), reinforcing its validity in assessing stress and negativity. The *other-focus* subscale (of which items are incorporated in our subscale of *commitment and responsibility*), was positively related to life satisfaction before (Reifman et al., 2007), suggesting that the capacity to focus on others is related to well-being.

Overall, we can conclude that these results tentatively support the theoretical concepts behind our subscales, and validate our previous description of the subscale *commitment and responsibility*.

### Limitations and future research

Future studies should research the applicability of the IDEA and thus Arnett’s theory of emerging adulthood more widely, both across cultures and in age range, as international studies show that his conceptualization of emerging adulthood cannot be singularly applied to other groups and cultures (Atak & Çok, 2008; Baggio et al., 2015; Dutra-Thomé, 2013; Hill et al., 2015; Leontopoulou et al., 2016; Lisha et al., 2014; Pérez et al., 2008). In addition, longitudinal studies to assess development of the subscales over time are needed to inform theory.

This specific study was also characterized by some limitations. To begin, both the exploratory factor analysis and the fitting of the resulting model were conducted using the same sample. In this way, we could evaluate whether the created model adequately represents the data in our sample, but not generalize this model across samples, which would further strengthen our findings, but would necessitate additional data. Furthermore, the skewed distribution of data led us to use nonparametric methods of analysis, which come with some limitations: analyses of variance (ANOVAs) can provide more concise results, and can be used to detect interaction effects. Nonparametric tests, however, remain more descriptive, and require additional interpretation with the aid of boxplots. We added effect sizes to aid interpretation of the Kurskall-Wallis tests, but their interpretation in the context of a nonparametric analysis is based on mean ranks instead of means, and thus less straightforward. In addition, we only found small effect sizes. As such, this manuscript stands as a first descriptive exploration of the topic. In addition, due to language differences, the questionnaires could not be distributed to French-speaking Belgians. As such, a replication study with a French translation of the IDEA should take place before our findings can be generalized to the whole of Belgium. We also did not collect data on ethnicity or cultural differences within our sample, which may play a role in development throughout young adulthood, to focus on the “general” Belgian culture first. In addition, the study’s cross-sectional design does not allow us to draw conclusions about developmental changes in terms of the IDEA-BE subscales. Accordingly, a longitudinal study assessing developmental trajectories of the IDEA-BE subscales could further solidify our findings. Finally, all questionnaires were self-reports, so that a portion of the observed variance could be shared method variance.

## Data Accessibility Statment

Raw data, analysis syntax, and materials used in this study are available upon request to the corresponding author.

## Footnotes

Using Pearson correlations between occupation, living situation, relationship status and the respective subscales while controlling for age, the proportion of covariance shared by occupation and the respective subscales that could be explained by age was explored. When controlled for age, none of the correlations between occupation, place of residence and relationship status remained significant at the .05 level, except the correlations between *commitment and responsibility* and occupation (*r* = .147, *p* = .001), and *commitment and responsibility* and relationship status (*r* = .229, *p* < .001), suggesting that the relationships between occupation and *commitment and responsibility*, and relationship status and *commitment and responsibility* are robust to age effects. This suggests some interaction between age and the other demographic factors, which can be further investigated in future studies. This publication was made possible through funding support of the KU Leuven Fund for Fair Open Access.
